# The clustering of diet, physical activity and sedentary behavior in children and adolescents: a review

**DOI:** 10.1186/1479-5868-11-4

**Published:** 2014-01-22

**Authors:** Rebecca M Leech, Sarah A McNaughton, Anna Timperio

**Affiliations:** 1 The Centre for Physical Activity and Nutrition Research (C-PAN), School of Exercise and Nutrition Sciences, Deakin University, 221 Burwood Hwy, Burwood 3125, Australia

**Keywords:** Cluster patterns, Diet, Physical activity, Sedentary behavior, Children, Adolescents, Socio-demographic correlates, Obesity

## Abstract

Diet, physical activity (PA) and sedentary behavior are important, yet modifiable, determinants of obesity. Recent research into the clustering of these behaviors suggests that children and adolescents have multiple obesogenic risk factors. This paper reviews studies using empirical, data-driven methodologies, such as cluster analysis (CA) and latent class analysis (LCA), to identify clustering patterns of diet, PA and sedentary behavior among children or adolescents and their associations with socio-demographic indicators, and overweight and obesity. A literature search of electronic databases was undertaken to identify studies which have used data-driven methodologies to investigate the clustering of diet, PA and sedentary behavior among children and adolescents aged 5–18 years old. Eighteen studies (62% of potential studies) were identified that met the inclusion criteria, of which eight examined the clustering of PA and sedentary behavior and eight examined diet, PA and sedentary behavior. Studies were mostly cross-sectional and conducted in older children and adolescents (≥9 years). Findings from the review suggest that obesogenic cluster patterns are complex with a mixed PA/sedentary behavior cluster observed most frequently, but healthy and unhealthy patterning of all three behaviors was also reported. Cluster membership was found to differ according to age, gender and socio-economic status (SES). The tendency for older children/adolescents, particularly females, to comprise clusters defined by low PA was the most robust finding. Findings to support an association between obesogenic cluster patterns and overweight and obesity were inconclusive, with longitudinal research in this area limited. Diet, PA and sedentary behavior cluster together in complex ways that are not well understood. Further research, particularly in younger children, is needed to understand how cluster membership differs according to socio-demographic profile. Longitudinal research is also essential to establish how different cluster patterns track over time and their influence on the development of overweight and obesity.

## Review

### Background

The unacceptably high prevalence of childhood overweight and obesity is a major public health issue in developed countries world-wide [1]. Obesity often persists into adulthood, increasing children’s risk of developing cardiovascular disease, type 2 diabetes and other chronic diseases later in life [2]. There is also good evidence that obesity is associated with high intake of energy-dense, nutrient poor (EDNP) foods such as soft-drinks, savory crisps, sweet biscuits and confectionary, and increased time spent in sedentary behaviors [3,4]. On the other hand, regular physical activity (PA) and a diet high in fruit, vegetables, legumes and whole-grain cereals have been shown to be protective [3,5].

Despite the importance of these modifiable factors in obesity etiology, there is evidence to suggest that many children and adolescents do not engage in these behaviors at the recommended levels (e.g. consume 2 serves of fruit and 5 vegetables [FV]/day, [6] achieve 60 mins of moderate-vigorous physical activity [7] or restrict screen-time to two hours or less/day [7]), with multiple obesity risk behaviors often occurring together. For example, Sanchez et al. [8] reported that almost 80% of 11–15 years olds had multiple dietary and PA risk factors. While in a representative population sample of 1,568 grade 6, 8 and 10 students from Australia, Hardy and colleagues found that approximately 51% boys and 43% of girls had three or more behavioral risk factors for obesity that included: low PA; high screen time; low FV; high soft drink consumption; and high snack intake [9].

Clustering or the co-existence of groups of people who share similar characteristics is a concept that has been successfully applied to understanding the relationships between different lifestyle behaviors [10,11]. The rationale underlying a focus towards clustering stems from the acknowledgement that the influences on lifestyle are multivariate and interactive [10]. For example, diet, PA and sedentary behavior may combine in complex ways that have a cumulative effect on the development of overweight and obesity [8]. This has important implications for public health because understanding which behaviors need to be targeted simultaneously and in whom obesogenic behaviors cluster together can be used to assist in the development of targeted obesity prevention initiatives. Interventions that are appropriately targeted and effectively bring about multiple behavior change, may be more cost-effective and maximise reach, to those most in need [12,13].

Exploratory, data-driven methods such as cluster analysis (CA) or latent class analysis (LCA) to investigate cluster patterns have become increasingly common. These approaches aim to maximally separate people into mutually exclusive groups who share similar characteristics [14,15]. Data-driven methodologies do not impose a predefined definition of what is a healthy or unhealthy behavior, and therefore they are a more person-centered approach that seeks to uncover homogenous groups of people based on the actual structure of the data [14]. For this reason, a number of studies have emerged in the past decade that have used these alternative methods to better understand the relationships between diet, PA and sedentary behavior among children and adolescents and a possible cumulative effect of an unhealthy clustering of these behaviors on the development of overweight and obesity [16-18]. However, no review of obesogenic cluster patterns derived using data-driven methods in children and adolescents could be found in the published literature. Childhood and adolescence are key periods of interest because these are potentially important times wherein patterns of obesogenic behaviors are shaped and evidence suggests that diet, PA and sedentary behaviors may track into adulthood [19-22]. In order to develop interventions for behavior change, we need to understand how these clusters differ by socio-demographic indicators. Age, gender and socio-economic status are of particular interest because these behaviors have been previously identified as important in influencing diet, PA and sedentary behaviors [23-26].

Therefore the aim of this paper is to critically review published studies in the peer-reviewed literature that have used data-driven methods to investigate the clustering patterns of diet, PA and sedentary behavior in children and adolescents and their associations with socio-economic indicators (age, gender and SES) and overweight and obesity.

### Methods

Searches were conducted by using Medline, PubMed, Science Direct, Scopus and Google Scholar Databases using the following key words and their alternatives (provided in parentheses) in their truncated forms (e.g. singular not plural noun forms): children, adolescents, clustering patterns (clusters, behavior patterns, lifestyle patterns) dietary behavior (diet, nutrition), physical activity (exercise, physical fitness) and sedentary behavior (sedentariness, sedentary lifestyle, television, inactivity). A manual inspection of the bibliographies of recent references was also undertaken. One reviewer screened the titles and the abstracts of the articles yielded from the searches to identify potentially-relevant articles. These articles were then examined in full to determine their eligibility for inclusion.

To be eligible for inclusion in this review, studies had to be published before November 2012, use data-driven methods (e.g. CA or LCA) and include school-aged children and adolescents (ages 5–18 years). All identified articles were checked and only those that included an examination of the “patterning”, “clustering” or “co-existence” of two or more obesogenic indicators of diet quality (e.g. FV consumption, EDNP food consumption, high-energy drinks, fat intake or overall diet quality); PA (intensity, duration or frequency during leisure-time and/or school-time), and sedentary behaviors (e.g. TV viewing, video watching, using the computer or internet and playing console games) were retained for this review. These behaviors are important correlates of overweight and obesity [4,5,27,28]. Studies that did not attempt to identify clusters on the basis of two or more of these behaviors or examined an interaction of these behaviors were not considered eligible.

While this review was not designed to be systematic, in order to provide a critical appraisal of literature and to identify potential gaps in the study of the clustering of obesogenic behaviors, the following characteristics of the studies were identified: 1) age range 2) sample size 3) study design 4) whether the reliability and validity of measures were reported 5) employment of strategies to minimise the subjectivity of data-driven methods to determine clusters, and 6) whether weight status was derived from self-reported or objective measures.

### Results

From the online literature search, 23 potential articles were initially retrieved. A further potential six articles were identified after manual inspection of several bibliographies. Of these articles, nine were excluded because focused on describing bivariate associations [29-31], co-prevalence of behaviors [8-10,32,33], or linear combinations (e.g. factors derived from factor analysis, not clusters) of behavior variables [34-36]. Eighteen articles (62% of potential studies) met the criteria for inclusion (Additional file 1). These studies were published between 2002 [37] and 2011 [18], of which 15 used CA [16,18,37-49] and three used LCA [17,50,51] to determine clusters. With the exception of one study [18] that included children aged 5–12 years, the ages of children and adolescents in these studies ranged from 9–21 years. The sample sizes were moderate to large, with only three studies using sample sizes <500 [18,45,48]. Most studies were conducted in Europe (9 studies [38-40,43-47,49]), or the USA (6 studies [16,17,41,42,50,51]), one was a cross-national study in the USA and UK [37], and one study was conducted in each of Australia [18] and Canada [48]. Most studies were cross-sectional, only four included a prospective study design [16,40,42,50].

### Cluster behaviors examined

Eight of the 18 studies examined the clustering of all three diet, PA and sedentary behavior [16-18,40,43,45,48,49], two studies examined diet and PA [41,44], and eight studies examined PA and sedentary behavior [32,37-39,42,46,47,50]. No study was found that has examined diet and sedentary behavior only. Six studies also included other health-related variables such as smoking and/or alcohol use [16,40,41,48], parental involvement [16,41], psychosocial behaviors [41,47] and dieting behaviors [16,17].

### Measurement of cluster behaviors

Only one study used 24-hour recall methodology to collect dietary data [43], two studies [18,51] used accelerometry to measure PA and one study [38] used ecological momentary assessment (EMA) to measure both PA and sedentary behavior. The remaining included studies relied on parental or self-report measures. Studies reporting both the reliability and validity for all of the diet, PA and sedentary behavior measures used were rare (Additional file 1).

### Methods to determine clusters

For studies that used CA, the methodological steps taken to ensure clusters were a reliable representation of the data were largely well described [14]. The influence that outliers and different absolute measurement scales of cluster variables may have on the cluster development were not addressed in three studies [39,41,49]. Only two studies [39,49] did not report the use of empirical procedures to minimise subjectivity in determining the number of clusters, and four studies [39,47-49] did not report the robustness of the final cluster solution. These issues are not relevant to LCA methodology which uses statistical modelling to indicate ‘best fit’ or number of clusters, also referred to as classes. For the three studies [17,50,51] that used LCA, the indices used to select the best fit model were clearly described.

### Cluster patterns

The number of clusters observed across the studies ranged from three [39,48,51] to seven [16,42]. The cluster pattern most frequently observed was a mixed PA/sedentary behavior pattern (8 studies [22,37,39,42,46,48,50,51]), characterised by either high levels of PA with high levels of sedentary behavior or vice-versa. No high diet quality/low sedentary behavior pattern was observed whereas a low diet quality/high sedentary behavior pattern was noted in three studies [17,18,40].

Two studies [41,44] found evidence of a healthy clustering of diet and PA, but the study by Mistry et al. [41] also included other health behaviors. In contrast, a low diet quality/low PA cluster was also reported in these same two studies. The healthy and unhealthy clustering patterns of diet and PA were observed despite the different methodologies used to measure the dietary behaviors; Sabbe et al. [44] calculated two dietary indexes (a ‘dietary diversity index’ and an ‘excess index’) from 19 food frequency questionnaire (FFQ) items, whereas Mistry et al. [41] used a measure of FV consumption from two short survey questions.

A common pattern across eight studies [37-39,42,46,47,50,51] examining the clustering of PA and sedentary behavior was that many clusters were defined by high levels of sedentary behavior. In a cross-sectional study of 1371 adolescents aged 13–16 years, Gorely et al. [38] identified five clusters for boys and girls separately, including an ‘Actives’ cluster and a ‘sedentary TV watchers’ cluster, based on six categories of PA and sedentary behavior that were measured in real time using EMA. In the latter cluster only 26% of boys and 9% of girls also met the PA recommendation of 60 minutes/day. Furthermore, the proportion of adolescents comprising the sedentary cluster was much greater than the proportion comprising the ‘Actives’ cluster (boys: 30% versus 15%; girls: 23% versus 14%).

Six studies [38,39,42,46,47,50] found cluster patterns characterised by high PA/low sedentary behavior, although two of these were large-scale prospective studies that both utilised National Longitudinal Study of Adolescent Health (Add Health) data but different methodologies (CA [42] and LCA [50]) to determine the clusters. Interestingly the CA and LCA approaches also yielded a different number of clusters with Nelson et al. [42] finding seven clusters and Liu et al. [50] finding five clusters. Three studies [46,47,51] found evidence to support an unhealthy clustering of PA and sedentary behavior, whereas other research suggests that high levels of PA can coexist with high levels of sedentary behavior or vice-versa [22,37,39,42,43,46,50,51].

All three diet, PA and sedentary behavior were found to cluster in healthy [18,43,45,49] and unhealthy ways [43,45,48,49]. However, most children and adolescents fell into a mixed category in which they exhibited one or two healthy behavior(s) concomitant with one or more unhealthy behavior(s). In a large cross-sectional study of 2084 adolescents from 10 European countries, Ottevaere and colleagues [43] identified an unhealthy, a healthy, and three mixed cluster patterns. The largest proportion of adolescents (42%) was categorized as inactive but with low levels of sedentary behavior and a high diet quality. Similar cluster patterns were found in Australian [18] and Belgian [45] child populations.

### Socio-demographic correlates of clusters

#### Age

Of seven studies [18,37,42,43,48,50,51] that investigated the association between cluster patterns and age, four [18,42,43,50] found that younger children/adolescents tended to be in clusters defined by high levels of PA, irrespective of the other behaviors examined. In two cross-sectional studies this relationship was gender specific with higher levels of PA observed in younger boys [51] and younger girls [37]. In the prospective “Add Health” study that examined self-reported PA and sedentary behavior in a nationally representative cohort of 13,339 American adolescents aged 11–21 years, Liu et al. [50] reported that older adolescents were significantly more likely to be in the low PA/low sedentary behavior cluster. Furthermore, for all clusters, the odds of engaging in ≥5 bouts of MVPA per day reduced into adulthood. The methodology of this study was strong; the rate of follow-up was high (88%) and adjustments were made for important confounders and baseline PA. In contrast, Turner et al. [48] found no association between age and clustering of PA with other behaviors, but their study used a convenience sample of adolescents of whom 85% achieved PA recommendations. Of two cross-sectional studies [18,43] that examined clustering of diet, PA and sedentary behavior, both found younger children were more likely to be in the healthiest cluster for these behaviors.

#### Gender

Nine of the 18 studies [17,39-45,48] examined the association between cluster patterns and age and seven studies [16,37,38,41,46,50,51] generated cluster patterns for boys and girls separately. All studies except one [48] found either different clusters patterns by gender or a significant difference in the proportion of boys and girls within the clusters. A consistent trend across these studies was that a higher proportion of boys were in the high PA clusters [37,39,44-46,50,51] whereas more girls were in the low PA clusters [38,39,41,46,50]. Common to three studies [43-45] was a healthy diet/low PA and a mixed or unhealthy diet/high PA cluster pattern that comprised more girls and boys, respectively. In contrast, Boone-Heinonen et al. [16] found an unhealthy clustering of diet and PA that was specific to girls.

Gender differences in cluster patterns have been found in studies that have examined a broader range of obesogenic behaviors. For example, Gorely et al.[38] and Marshall et al. [37] observed gender specific clusters where girls tended to engage in higher levels of homework and/or socializing on the phone whereas boys were more likely to be in the high screen-time clusters. Liu et al. [50] found that males were more likely to be in a high PA cluster where ‘skating’ was the dominant sport. Huh et al. [17] reported two high sedentary behavior/high fat and sugar snack clusters that were differentiated by being either weight conscious or not weight conscious, with girls significantly less likely than boys to be in the latter cluster relative to an “active, healthy eating” cluster (Odd ratio [OR] = 0.34, p = 0.005).

### Socio-economic status

Nine studies were identified that examined whether cluster patterns could be characterised by SES [16,17,40,41,43,44,50-52]. Level of parental education was the most common measure of SES [16,41,43,44,50-52], followed by parental income [16,42,50]. Other measures of SES included: receipt of free or reduced cost school lunches [17], poverty level [41] and adolescent education level (according to Germany’s different types of school systems) [40]. Cluster patterns characterised by high PA/sports participation were significantly associated with a higher level of parental education [16,50,52], family income [16,42,50] and adolescent education level [40], regardless of the other behaviors examined and even after controlling for gender [16,50]. High sedentary behavior clusters were associated with low parental education, family income [16,42,50] and receipt of free/reduced cost school lunches [17]. Additionally, Ottevaere et al. [43] reported that boys and girls who have the higher educated parents were more likely to be in the healthy cluster and the healthy eating, low PA and low sedentary behavior cluster. Conversely, two studies found no association between cluster membership and SES [44,51].

### Associations with overweight/obesity

Additional file 2 presents a table that summarises the 13 studies identified that have examined associations between cluster membership and weight status or body mass index (BMI). Weight status or BMI was calculated from measured height and weight in seven studies [16-18,39,40,43,51]. Most studies [37,40,43-46] used internationally accepted cut-points [53] to determine weight status while three studies [16,17,51] used USA specific cut-points [54]. Eight studies [18,37,39,40,43-45,48] employed univariate or bivariate statistical techniques to investigate associations and few [16,40] examined associations longitudinally. Of these, five, including two longitudinal studies, found evidence of a possible synergistic effect of multiple unhealthy behaviors on overweight or obesity [16,17,40,46,51], two studies found an unexpected inverse association with an unhealthy cluster pattern [16,49], and seven studies found no association [18,37,39,43-45,48].

Across four studies, clusters characterised by either low PA [16,40,46] or high sedentary behavior [16,17,46,51] were positively associated with overweight. In a large cross-sectional study (n = 12,538) based in 9 European countries, te Velde et al. [46] found that compared to children in the high PA/low sedentary behavior cluster, boys and girls in the low PA/high sedentary behavior cluster and the high TV viewing cluster, had the highest odds of being overweight, respectively. This study was of high quality with high response rates and adjustment for important confounders. Huh et al. [17] reported significantly higher obesity prevalence among 997 4th grade children in two clusters defined by high TV/video game use, consumption of high fat, high sugar snacks compared to three other clusters (p < 0.001). Conversely Cameron and colleagues [18] observed no association between BMI z-scores and the “Energy-dense snackers who watch TV cluster”. However, in the study by Huh et al. [17], 41% of the sample was overweight or obese and Cameron et al. [18] drew their sample from areas of low SES, limiting the generalizability of these results.

Bivariate analyses of a four year longitudinal study conducted in a subsample (n = 389) of German children aged ten at baseline found obesity incidence was significantly higher (p < 0.05) in the low PA/mixed diet quality/moderate media time cluster [40]. One large prospective study (n = 9,251) of 11–21 year olds found, that compared to the high PA/sports cluster, adolescent girls in the: ‘Average diet & activity’, ‘Sedentary behaviors’, and ‘Restrictive dieting & smoking’ clusters were more likely to be obese five years later, after adjusting for race, household income, parental education, age and season [16]. No longitudinal associations were observed among boys in this study.

### Discussion

This review of studies that have used data-driven methodologies to examine clustering of obesogenic behaviors has shown that diet, PA and sedentary behavior cluster in children and adolescents in both healthy and unhealthy ways. It has also demonstrated that cluster patterns are complex with all studies observing the co-occurrence of both healthy and unhealthy behaviors. This review found a high frequency of clusters defined by high levels of sedentary behavior. This is of concern given the growing evidence to suggest that sedentary behavior is independently and positively associated with poor health outcomes [55,56]. Cluster patterns were found to differ according to age, gender and SES. A consistent finding was the higher proportion of girls; older children/adolescents; and children/adolescents from a low SES in the clusters defined by low levels of PA. There was some evidence to suggest that boys are more likely to be in clusters characterized by poor diet quality and that children from a low SES are more likely to be in clusters defined by high levels of sedentary behavior; however it should be noted that these conclusions are based on a small number of studies that were inconsistent in the types of dietary, PA and/or sedentary behaviors examined. The evidence in relation to a cumulative effect of these behaviors on obesity outcomes is inconsistent; some studies found a higher prevalence of overweight/obesity in unhealthy clusters while other studies found no association at all. However, the consistent finding that older children/adolescents and girls are over-represented in clusters defined by low PA is supported by a wealth of data that consistently shows PA to decline with age [22,57-59] and that girls are less active than boys [57,60,61].

### Limitations of the reviewed studies

It has been previously suggested that CA may be a potential tool for screening populations because it reveals what behaviors actually coexist within populations [43,44]; however this statistical method, like LCA, is not without its limitations. Importantly, the cluster patterns observed may only be specific to the cultures and populations studied and caution is needed when generalizing results from studies that use data-driven methods. CA involves subjective decision-making by the researcher when deciding: 1) whether to determine gender or age-specific clusters; 2) how the cluster variables are treated (e.g. continuous or categorical); 3) which type of clustering method to use (e.g. Ward’s method or k-means); and finally, 4) which cluster solution is the most “meaningful” - all of which can affect the number and types of clusters determined [14]. LCA has some advantages over CA because this approach minimises subjectivity by using statistical indexes to indicate the best model fit or number of clusters [15]. However, it is important to note that the choice of “best model fit statistics” is also subjective, varies across studies and may therefore influence cluster identification. LCA may also yield different results to CA. For example, two studies analysed the same data, one using CA [42] and the other LCA [50], but found a different a number of clusters. While most studies adequately reported how the clusters were developed and assessed cluster reliability, there was substantial heterogeneity in the types of clustering methods used and how cluster variable were treated across these studies, which may partly explain the inconsistencies in the types of clusters found.

Most of the studies included in this review were cross-sectional. This is an important limitation because children and adolescents who are overweight or obese may be actively trying to lose weight therefore confounding important associations. Currently it is unclear whether obesogenic cluster patterns are longitudinally associated with different socio-demographic indicators and overweight and obesity. Furthermore the stability of cluster patterns over time has not been explored. The types of dietary, PA and sedentary behaviors examined across the studies were heterogenous, making studies difficult to compare. Cluster patterns tended to differentiate more strongly for PA and sedentary behavior than for dietary behaviors; however almost all the studies included the examination of PA and sedentary behavior and just over half of the studies examined dietary behaviors. Few studies have specifically examined the clustering of diet and PA and no study could be found that has examined the clustering of diet and sedentary behavior. The dietary behaviors examined across the studies were inconsistent and highly variable which limits comparisons across studies.

While the scope of this review was limited to school-aged children and adolescents (age-range: 5–18 years), the majority of studies identified focused on older children or adolescents aged nine years and over. Approximately half of the studies reviewed investigated the socio-demographic correlates of cluster patterns, making it difficult to draw definitive conclusions about associations. In addition, indicators of SES, other than parental education, have been little researched.

Another limitation of the included studies is that self-report surveys were used to collect data on the examined behaviors with a number of studies not reporting their reliability and validity, particularly for the sedentary behavior variables. Where reported, most instruments had acceptable reliability but low to moderate validity. Only one study measured diet using a 24 hour recall, two studies used an objective measure of PA, and no study used an objective measure of sedentary behavior. The recall and social-desirability biases that accompany self-report measures are well known and may lead to misclassification bias [62,63]. This issue was highlighted by Saunders et al. [64] who noted that the study by Jago et al. [39] reported large differences between clusters using the self-report PA measures when the accelerometer data showed little difference between the groups. This issue may also be compounded in six studies that examined associations with overweight/obesity based on self-report measures of weight status. While Goodman et al. [65] concluded that BMI based on adolescent self-report height and weight is a valid measure of weight status, some evidence suggests that parental report may overestimate children’s weight [66,67] and underestimate children’s height [67]. As for dietary intake, underreporting of energy intake by children and adolescents has been consistently positively associated with adiposity, and increases with age, but the impact of children’s weight status on parental report of children’s dietary intake is currently unclear [68]. While robust and valid measures are critical for accurate assessment of diet, PA and sedentary behavior, it is important to recognise that each life-stage (young childhood, older childhood and adolescence) presents unique challenges to researchers in the selection of measurement tools that are not only feasible but can accurately capture these behaviors [69,70].

### Strengths and limitations of review

This review was the first to examine the clustering of diet, PA and sedentary behavior in children and adolescents, and their associations with socio-demographic indicators and overweight/obesity. Studies were identified from an extensive search of the published literature conducted in a range of data-bases. The broad definition of search terms applied across multiple databases enabled the identification of many potential studies; however, as this was not a systematic review, it is possible that some studies may have been missed. This review focussed on a limited range of diet behaviors (e.g. intake of FV, EDNP foods or high energy drinks and measures of overall diet quality), socio-demographic indicators (age, gender and SES), and included only studies that have used data-driven methodologies, therefore narrowing its scope. This review also included studies that have examined other health behaviors such as smoking or alcohol. It is unclear whether these behaviors influenced the types of clusters generated and whether this influenced the relationship between obesogenic behaviors and socio-demographic characteristics and obesity outcomes. Nonetheless, this review may provide important insights into how obesogenic behaviors cluster together and in whom they cluster together, that may assist the design and targeting of public health initiatives aimed at combating obesity.

### Conclusions: Implications for future research

This review of studies that have used data-driven methodologies to examine clustering of obesogenic behaviors has shown that diet, PA and sedentary behaviors can cluster in complex ways that are both conducive and deleterious to good health and that these clusters differ across socio-demographic groups and are not consistently associated with overweight/obesity. The complexity of these findings implies that we cannot assume that healthy levels of one behavior is indicative of an overall healthy lifestyle. Healthy behaviors may potentially compensate unhealthy ones and explain the equivocal association with overweight/obesity. This suggests that screening of populations may help to appropriately select and target behaviors for obesity prevention, which may in turn maximise the reach and cost-effectiveness of these interventions [13].

Of concern, is the number of clusters defined by high levels of sedentary behavior with some evidence suggesting important gender differences in the types of sedentary behaviors pursued by children and adolescents [37,38]. Future research should consider examining how different types of sedentary behavior cluster with diet and PA for boys and girls separately. The identification of older children and adolescents, and particularly females, in the clusters defined by low PA, suggest that research designed to elucidate the mediators of low PA in these target groups would be beneficial.

Given the consistent finding of age and gender differences in the types of obesogenic cluster patterns found among children and adolescents, future research should incorporate samples sizes sufficient to determine cluster patterns separately by age and gender. There is also a need for clusters to be determined from valid and reliable measures of diet, PA and sedentary behavior; however, additional evidence examining a broader range of behaviors and their contextual and/or environmental mediators would also provide rich information to inform future interventions.

Longitudinal evidence that examines younger children is crucial; understanding how the clustering of obesogenic behaviors track over time and the critical periods where PA declines and poor dietary habits and sedentary behavior increase during childhood are imperative to inform the timing of interventions. Large-scale prospective studies are also needed to establish how cluster patterns vary by different socio-demographic indicators and the long-term exposure of different clustering patterns on the development of overweight and obesity.

## Abbreviations

BMI: Body mass index; CA: Cluster analysis; EMA: Ecological momentary assessment; EDNP: Energy-dense nutrient poor; FFQ: Food frequency questionnaire; FV: Fruit and vegetable; LCA: Latent class analysis; OR: Odd ratio; PA: Physical activity; SES: Socio-economic status; TV: Television.

## Competing interests

All authors declare that they have no competing interests.

## Authors’ contributions

RML retrieved and critically analysed the studies within the review. RML drafted the manuscript. AT and SAM supervised the study and assisted with the analysis and interpretation of the reviewed studies. All authors critically reviewed drafts of the manuscript. All authors read and approved the final manuscript.

## Authors’ information

RML was an Honours student at Deakin University at the time the manuscript was drafted and is currently a Ph.D. student, also at Deakin University. RML is a registered Associate Nutritionist (ANutr). RML’s qualifications also include a Bachelor of Food Science & Nutrition (Honours) and a Master of Clinical Epidemiology (coursework). AT (Ph.D.) is an Associate Professor and Senior Researcher at Deakin University. SAM (Ph.D.) is an Australian Research Council Future Fellow and an Accredited Practising Dietitian (APD) at Deakin University.

## Supplementary Material

Additional file 1 Characteristics of studies included in review of clustering of diet, PA and sedentary behaviors of children and adolescents*. Click here for file

Additional file 2 Associations of clustering patterns of diet, PA and sedentary behaviors with BMI or weight status in children and adolescents. Click here for file
