# Supplementary material for: The clustering of diet, physical activity and sedentary behavior in children and adolescents: a review
Source: Int J Behav Nutr Phys Act. 2014 Jan 22;11:4. doi: 10.1186/1479-5868-11-4 (PMC3904164; doi:10.1186/1479-5868-11-4)
Supplement: Additional file 1 — Characteristics of studies included in review of clustering of diet, PA and sedentary behaviors of children and adolescents*. [file 1479-5868-11-4-S1.pdf]

Additional file 1 Characteristics of studies included in review of clustering of diet, PA and sedentary behaviors of children and adolescents*

First author (year)	Country	Design (C/S or P)	Sample Size	Age (years)	Cluster pattern method	Diet cluster variables	Diet assess. method	PA cluster variables	PA assess. method	SB cluster variables	SB assess. method	Socio-demographic variables (A/G/SES)
Boone-Heinonen (2008)[16] ǂ	USA	P	9,251	11-21	CA (B/G)	Diet beverages, regular beverages, juice, fruit, vegetables, low-fat foods, meat, added fat, sweets, fried food, dairy items, vitamins, fast food meals, meals/day, meals with parents, makes own decision about food.	FFQ, Q	Housework, hobbies, skating, sports, exercise, hanging out, school clubs, school team sports, school individual sports, PE classes, sports with parents, use recreation centre, exercise to lose weight	7-day recall Q[1,2]	TV, *v*ideos, computer or video games, makes own decisions re TV	7-day recall Q	A, SES
Cameron (2011)[18]	Australia	C/S	352	5-12	CA	FV, EDNP food and drink	FFQ (PR)	Time spent in MVPA	Accel.[1,2]	Screen-time (TV,DVDs,console games, computer games, computer or internet)	Q (PR)	A
Gorely (2007)[38]	UK	C/S	1,371	13-16	CA (B/G)	NA	NA	Sports/exercise, paid work/chores	4-day diary (EMA)[1]	TV or videos, computer use, socialising behaviors	4-day diary (EMA)[1]	A

										(hanging out, sitting, talking, phone), homework		
Huh (2011)[17]	USA	C/S	997	9.28 ±0.48	LCA	Salty high-fat snack foods, high-sugar snack foods, soda, fruit, vegetables	Q	Hard PA, team sport involvement	Q	Screen time (TV and video games)	Q	G, SES
Jago (2010)[39]	UK	C/S	761	10-11	CA	NA	NA	School sports clubs at school, sports clubs outside of school, playing with friends near home, playing in home or garden	Q	TV viewing, using computer (excl. homework), using phone/texting, console games	Q[2]	A, G
Landsberg (2010)[40] ǂ	Germany	C/S & P	1,894 & 389	14 & 10-14	CA	3 dietary patterns (Healthy, risk related and mixed)	26-item FFQ[1,2]	Structured LTPA, unstructured LTPA, mode of transport to school	Q[1]	Media time	Q[1]	G, SES
Lui (2010)[50]	USA	P	13,339	Grades 7-12	LCA (B/G)	NA	NA	Housework, hobbies, skating/biking, active sports, exercise	7-day recall Q[1,2]	Screen time (TV, videos, *c*omputer or video games, *c*omputer)	7-day recall Q[(1-TV only)]	A, SES

Marshall (2002)[37]	USA & UK	C/S	2,494	11-15	CA (B/G)	NA	NA	Total PA (32 types of PA)	SAPAC[1,2]	TV, computer or internet, video games, homework, reading, sit & talk, telephone	SAPAC[1]	A
Mistry (2009)[41] ǂ	USA	C/S	4,010	14.45	CA (B/G)	FV	Q	MVPA	Q [1,2]	NA	NA	A, SES
Nelson (2005)[42]	USA	P	11,957	Grades 7-12	CA	NA	NA	Housework, hobbies, skating/biking, active sports, exercise, school PE, school academic clubs, school team sports, school individual sports, sport with parents, recreation centre use	7-day recall Q[1,2]	TV, videos, computer or video games, computer, makes own TV decisions	7-day recall Q[(1-TV only)]	A, G,SES
Ottevaere (2011)[43]	10 from Europe	C/S	2,085	(I) 12.5-14.99<br>(II) 15-17.5	CA	Dietary quality Index for Adolescents (DQI-A)	24hr recall[1,2]	MVPA	IPAQ-A[1,2]	Time in SB (TV, computer games, console games, internet, internet for study,	Q[1]	A, G, SES

										studying or homework)		
Patnode (2011)[51]	USA	C/S	720	14.7	LCA (B/G)	NA	NA	MVPA weekday, MVPA weekend<br><br>Traditional sports, fitness activities, other sports or playing, chores or work	Accel.[1,2]<br><br>3DPAR[1,2]	TV, DVDs or videos, video or computer games, internet or computer, talking/texting on phone, reading or homework	Q	A, SES
Sabbe (2008)[44]	Belgium	C/S	1,725	10	CA	Dietary diversity index, Excess index	19-item FFQ[1,2]	MVPA	FPAQ[1,2]	NA	NA	G, SES
Seghers (2010)[45]	Belgium	C/S	317	11-12	CA	Healthy food index, Risk related food index	FFQ[1,2]	LTPA	FPAQ[1,2]	LT screen-based media (TV, videos and recreational computer, console games)	Q	G
te Velde (2007)[46]	9 from Europe	C/S	12,538	9-14	CA (B/G)	NA	NA	Moderate-to-vigorous LTPA	Q[1]	TV , computer	Q[1]	-
Telama (2005)[47] ǂ	Belgium & Finnland	C/S	1439 & 789	12-15	CA	NA	NA	Organized & unorganized PA, participation in competitions	Q[1,2]	Computer games, TV, video	Q[1,2]	G

Turner (2011)[48] ǂ	Canada	C/S	445	14-17	CA	FV	WHO STEPS Q	MVPA	SAPAC [2]	TV or videos, computer or internet, talking with friends (incl. phone)	SAPAC	A, G
van der Sluis (2010)[49]	Norway	C/S	884	10-17	CA	FV, snacks and soda (chocolate, candy, potato chips, soda)[1,2]	Q	Leisure time MVPA	Q	TV or computer	Q	-

Abbreviations: 3DPAR, 3-day physical activity recall; 24hr, 24 hour dietary recall; A, age; accel., accelerometry; A/G, analysis stratified by age group; assess., assessment; B/G, boys and girls analyzed separately; CA, cluster analysis; C/S, cross-sectional; excl., excluding; EMA, ecological momentary assessment; EDNP, energy-dense, nutrient poor, FFQ, food frequency questionnaire; FPAQ, Flemish Physical Activity Questionnaire; FV, fruit and vegetables; G, gender; incl., including; IPAQ-A, International Physical Activity Questionaire (7-day recall); LCA, latent class analysis; LTPA, leisure time physical activity; MVPA, moderate-to-vigorous physical activity; NA, not assessed; PA, physical activity; Q, questionnaire; PR, parental report; SAPAC, Self-administered physical activity checklist; SES, socio-economic status; P, prospective; PE, physical education; SB, sedentary behavior; WHO, World Health Organization.

[1]Values for reliability reported or could be found in the reference(s) provided
[2]Values for validity reported or could be found in the reference(s) provided
*All studies included both boys and girls
ǂAlso examined other behaviors (e.g. alcohol, smoking, dieting and psychosocial behaviors)
