# Supplementary material for: The clustering of diet, physical activity and sedentary behavior in children and adolescents: a review
Source: Int J Behav Nutr Phys Act. 2014 Jan 22;11:4. doi: 10.1186/1479-5868-11-4 (PMC3904164; doi:10.1186/1479-5868-11-4)
Supplement: Additional file 2 — Associations of clustering patterns of diet, PA and sedentary behaviors with BMI or weight status in children and adolescents. [file 1479-5868-11-4-S2.pdf]

Additional file 2 Associations of clustering patterns of diet, PA and sedentary behaviors with BMI or weight status in children and adolescents.

Study [#]	Obesogenic patterns by behaviors examined	BMI or weight status[ǂ]	Method of analysis	Results
Diet and PA				
[44]	<u>5 clusters</u><br>1. Sporty Healthy Eaters<br>2. Sporty Mixed Eaters<br>3. Moderate Active Healthy Eaters<br>4. Unsporting Unhealthy Eaters<br>5. Sedentary Healthy Eaters	Weight status	χ2 test stratified by gender	No association
PA and sedentary behaviour				
[39]	<u>3 clusters</u><br>1. High active/low sedentary<br>2. Low active/moderate sedentary<br>3. High active/low sedentary	BMI z-score*	ANOVA	No association
[37]	<u>3 clusters (boys)</u><br>1. Techno actives<br>2. Non-socializing actives<br>3. Uninvolved inactives<br><u>3 clusters (girls)</u><br>1. Sociable actives<br>2. Non-socializing actives<br>3. Uninvolved inactives	Weight status (categorised as underweight, normal weight, overweight, obese)	χ2 test	No association

[51]	3 classes (boys)<br>1. Active<br>2. Sedentary<br>3. Low media/moderate activity<br>3 classes (girls)<br>1. Active<br>2. Sedentary<br>3. Low media/functional activity	Weight status* (categorised as overweight)	Latent multinominal logistic regression	A higher proportion of girls in class 2 were overweight compared to classes 1 and 3 ($p<0.05$). A higher proportion of boys in class 2 were overweight compared to class 1 ($p<0.05$).
[46]	5 clusters (boys)<br>1. Healthy behavior pattern<br>2. High TV viewers<br>3. Mixed pattern<br>4. High PC users<br>5. Unhealthy behavior<br>5 clusters (girls)<br>1. Healthy behavior<br>2. High TV viewers<br>3. Low sedentary behavior and low physical exercise<br>4. High PC users<br>5. Unhealthy behavior	Weight status	Sex stratified multilevel logistic regression adjusted for age. Random intercept assigned for country and school level.	Compared to cluster 1, boys in clusters 3 (OR=1.5; 95% CI: 1.18-1.90) cluster 4 (OR=1.43 ; 95% CI: 1.05-1.96) and cluster 5 OR=1.18 ; 95% CI: 1.18-2.37) and girls in cluster 2 OR=1.64 ; 95% CI: 1.23-2.18) and 4 OR=1.42 ; 95% CI: 1.00-2.01) were more likely to be overweight.
Diet, PA and sedentary behaviour				
[16]ǂ	7 clusters (boys)<br>1. School Clubs & Sports<br>2. Sports<br>3. Moderately active<br>4. Sedentary Behaviors<br>5. Junk Food & Smoke	Weight status (obesity prevalence, 5 year obsesity incidence)*	Sex stratified multivariate logistic regression adjusted for race, household income, parental	Compared to cluster 1, clusters 2, 4 and 6 were significantly associated with obesity prevalence and incidence among girls. Among boys, being in cluster 4 was inversely associated with obesity

	6. Dieters<br>7. Low diet & activity<br><br><u>clusters (girls)</u><br><br>1. School Clubs & Sports<br>2. Average diet & Activity<br>3. High Consumer<br>4. Sedentary Behaviors<br>5. Junk Food & Low Activity<br>6. Restrictive Dieting & Smoking		education, region and wave specific age and season.	prevalence but not obesity incidence, compared to cluster 1.
[18]	<u>5 clusters</u><br><br>1. Young PA enthusiasts<br>2. All-round healthy behaviors<br>3. Screen-time focussed<br>4. Low on FV and PA<br>5. Energy dense eaters who watch	BMI z-score*	ANOVA	No association
[17]‡	<u>5 classes</u><br><br>1. High-sedentary, high-fat/high-sugar snacks, not weight conscious<br>2. High-sedentary, high-fat/high-sugar snacks, weight conscious<br>3. Dieting without exercise, weight conscious<br>4. Active, healthy eating<br>5. Low healthy, snack food, inactive, not weight conscious	Weight status* (categorised as obese, overweight, healthy, underweight)	$\chi 2$ test	Higher overweight and obesity prevalence in classes 1 and 2 ($\chi 2=279.8$, $p<0.001$)
[40]‡	<u>3 clusters</u><br><br>1. Low activity, low-risk behavior<br>2. High media time and high-risk behavior<br>3. High activity, high-risk behavior	Overweight and obesity prevalence*<br>Four year incidence of overweight/obesity*	$\chi 2$ test	Four year incidence of obesity highest in cluster 1 and lowest in cluster 3 ($p<0.05$). No other associations were found with weight status.

[43]	5 clusters<br>1. Unhealthy<br>2. Sedentary<br>3. Active, low diet quality<br>4. Inactive, high diet quality<br>5. Healthy	Weight status* (categorised as underweight, normal weight, overweight, obese)	χ2 test stratified by gender	No association
[45]	4 clusters<br>1. Sport media-oriented mixed eaters<br>2. Academic healthy eaters<br>3. Inactive healthy eaters<br>4. Inactive media oriented unhealthy eaters	Weight status	ANOVA stratified by gender	No association
[48]‡	3 clusters<br>1. Active, high screen-time users<br>2. Active, low screen-time users<br>3. Less active, least frequent drinkers	BMI z-scores	ANOVA	No association
[49]	4 clusters<br>1. Healthy<br>2. Quite healthy<br>3. Quite unhealthy<br>4. Unhealthy	BMI (kg/m^2)	Linear regression adjusted for gender and parental education	Being in cluster 4 (compared cluster 1) was inversely associated with BMI (β=-1.27, p=0.048)

Abbreviations: BMI, body mass index; CI, confidence interval; FV, fruit and vegetable; PA, physical activity;

* Derived from measured height and weight

ƚ Unless indicated, weight status categories included overweight (including obesity) or not overweight

‡Also examined other behaviors (e.g. alcohol, smoking, dieting and psychological behavior)
